# YOLO-Crack: Geometry-Guided Real-Time Crack Detection Framework Toward Edge Deployment

**DOI:** 10.3390/s26123892

**Published:** 2026-06-18

**Authors:** Zhe Wei, Rui Wang, Rong Dai, Haibo Xu, Huan Zhang, Yurong Zou

**Affiliations:** 1 School of Computer Science and Artificial Intelligence, Civil Aviation Flight University of China, Guanghan 618307, China; findz@cafuc.edu.cn (Z.W.); huanzhang@cafuc.edu.cn (H.Z.); amy0915@cafuc.edu.cn (Y.Z.); 2 Guanghan Flight College, Civil Aviation Flight University of China, Guanghan 618307, China; wave@cafuc.edu.cn

**Keywords:** crack detection, YOLOv11, attention mechanism, geometric constraints, TensorRT deployment

## Abstract

Crack detection in mobile inspection scenarios is constrained by both the extremely slender geometry of crack targets and the real-time inference requirements on edge devices, which expose systematic limitations of general-purpose object detectors. This paper proposes YOLO-Crack, a closed-loop solution that couples geometry-statistics-driven module design with end-to-end edge deployment validation. On the algorithmic side, we first quantify crack geometric properties and then introduce (i) a crack-aware cross-dimensional fusion attention (CFCA) module to strengthen feature representations, (ii) a dual-path feature enhancement module (DFEM) to preserve fine details during upsampling, and (iii) an empirical smooth quality window adjustment with shape consistency regularization to stabilize bounding-box regression for slender cracks. Experiments on the Crack500 dataset show that YOLO-Crack achieves 78.8% precision, 51.4% recall, and 65.7% mAP@0.5, improving over the YOLOv11n baseline by 4.2, 1.7, and 2.9 percentage points, respectively. On the engineering side, we deploy YOLO-Crack on a Jetson Orin NX mobile robot platform and evaluate it in a real ROS pipeline; the measured end-to-end throughput reaches 25.5 FPS, meeting real-time video processing requirements. The proposed framework provides a practical reference workflow for edge vision tasks, from geometry analysis to engineering verification.

## 1. Introduction

Infrastructure is the material foundation supporting economic development and social operation, and its safety is directly related to public security and societal well-being. With the rapid pace of urbanization, a large number of concrete structures (such as bridges, tunnels, and dams) inevitably suffer from aging and damage during long-term service. As one of the most common early-stage defects in concrete structures, cracks not only degrade load-bearing capacity and durability, but may also trigger secondary hazards such as reinforcement corrosion and leakage, and can even lead to catastrophic failures, including structural collapse [1,2]. It has been reported that the global economic loss caused by inadequate infrastructure maintenance reaches hundreds of billions of dollars annually, making timely and accurate crack detection and early warning a critical component for ensuring structural safety and extending service life [3].

Conventional manual inspection is limited by low efficiency, high labor cost, and strong subjectivity, and it also poses safety risks when inspecting hard-to-reach areas. These inherent drawbacks hinder its large-scale adoption in modern structural health monitoring (SHM) systems [4,5]. Consequently, unmanned aerial vehicles (UAVs) and ground mobile robots equipped with vision-based inspection systems have gradually become mainstream solutions for automated inspection. However, due to the constrained computational resources and power budgets of edge devices, achieving accurate real-time processing of captured images under tight resource constraints remains a key bottleneck for practical deployment [6,7].

In recent years, deep learning has achieved remarkable progress in computer vision, offering new technical routes for crack detection in mobile inspection scenarios. In particular, single-stage object detectors represented by the YOLO (You Only Look Once) family unify detection as a regression problem and deliver millisecond-level inference while maintaining competitive accuracy, making them well-suited for real-time inference on edge platforms [8]. As a recent generation of the YOLO architecture, YOLOv11 further improves feature extraction and multi-scale fusion through an enhanced C3k2 module, strengthened Spatial Pyramid Pooling Fast (SPPF), and an optimized decoupled detection head, providing an efficient baseline for resource-limited crack detection tasks [9].

Nevertheless, directly applying general-purpose detectors to edge-based mobile inspection still faces a dual challenge arising from target characteristics and resource constraints. First, cracks often exhibit slender and elongated shapes, diverse orientations (horizontal, vertical, oblique, or intersecting), low-contrast boundaries, and strong susceptibility to interference from textured backgrounds [10,11]. These properties require stronger geometric awareness and better detail preservation from the detector [12]. Second, mobile and edge platforms impose strict requirements on real-time performance and resource efficiency, necessitating accurate inference under limited compute and power budgets [13].

To address these challenges, a growing body of research has explored feature-representation enhancement to achieve a better accuracy–efficiency trade-off under task-specific constraints. On the one hand, structural re-parameterization and lightweight convolution designs are widely adopted to reduce computational cost. Su et al. [14] proposed MOD-YOLO, introducing a depthwise separable convolution (MODSConv) that preserves the original dimensionality to retain channel information, achieving good accuracy with substantially fewer parameters. Zhu et al. [15] integrated hybrid attention and residual blocks to construct an extremely lightweight encoder–decoder network with only 0.57 M parameters. On the other hand, to cope with the variability of crack morphology, dynamic receptive fields and feature-fusion strategies have become key directions. Yang et al. [16] introduced deformable convolution (DCN) in Flexi-YOLO to adaptively model non-linear crack patterns; Ren et al. [17] enhanced the Path Aggregation Network (PAN) by adding weighted fusion to strengthen multi-scale interactions; and Zhu et al. [18] proposed a dual-stream backbone from a frequency-domain perspective to process texture and semantics in parallel. These methods improve the accuracy–efficiency trade-off from different perspectives; however, flexible operators may introduce additional deployment overhead, whereas aggressive lightweight compression can weaken the representation of subtle crack details [19], indicating that fine-detail preservation and edge efficiency remain difficult to balance [20,21].

Attention mechanisms constitute another active research direction, aiming to make models focus on crack regions while suppressing background noise. Duan et al. [22] and Appe et al. [23] integrated SE-Net and CBAM, respectively, in underwater pipeline and tomato crack detection, demonstrating the effectiveness of channel/spatial reweighting in background suppression. Huang et al. [24] introduced Triplet Attention (TA) to strengthen channel–spatial interactions, while Yin et al. [25] explored large-kernel attention to capture long-range dependencies. Considering crack morphology, some studies further introduced position-sensitive designs. For example, Xing et al. [26] and Chen et al. [27] used Coordinate Attention to decouple feature aggregation along horizontal and vertical directions.Although these attention mechanisms improve feature saliency, most of them were originally designed for generic visual recognition or compact-object detection [28,29]. They usually enhance channel, spatial, or coordinate responses, but do not explicitly model the line-like, anisotropic, and direction-continuous geometry of cracks [30]. This may lead to fragmented responses when cracks are oblique, curved, intersecting, or embedded in complex textured backgrounds.

Loss function design also plays a crucial role in allocating optimization pressure among positive samples. To address class imbalance and the distribution of easy vs. hard samples in crack detection, several improvements have been proposed. Yu et al. [31] and Ding et al. [32] employed Focal Loss and EIoU loss to reweight classes and improve regression convergence, respectively. Chen et al. [27] proposed ECIOU-Loss, and Huang et al. [24] designed FP-IoU to balance detection speed and bounding-box regression. Although these strategies improve performance on benchmark datasets, many of them either rely on explicit sample reweighting or remain dominated by the global overlap score, and they do not fully address the weak sensitivity of IoU-based losses to short-side deviations in large-aspect ratio crack boxes. This motivates a more conservative empirical adjustment: preserving the original CIoU objective while adding a smooth quality window term and shape consistency constraints to calibrate regression supervision for slender targets.

Although the above studies have made progress at the module level, most existing work focuses on offline metric improvements and lacks systematic, closed-loop validation from algorithm design to edge deployment. Some studies focus on system integration. Sagar et al. [33], Qiu et al. [34], and Alkhedher et al. [35] developed robotic or UAV-based automated acquisition platforms, while Zhang et al. [36] and Guo et al. [37] explored specific applications such as crack sealing robots and high-speed railway girder inspection. However, these system-level studies typically reuse generic ResNet- or YOLO-based detectors without hardware-aware optimization, resulting in high on-device latency or reliance on offline post-processing. Other studies focus on lightweight algorithm design. Various efforts have explored pruning, architecture search, and quantization to facilitate edge deployment [38,39,40,41]. However, these approaches often trade feature representation for speed, leading to high miss rates for subtle cracks. More importantly, few of them jointly consider crack-specific geometric priors or fully leverage hardware acceleration frameworks such as TensorRT [42,43,44]. As a result, models with low theoretical FLOPs may still fail to meet the real-time requirements of closed-loop mobile robot control in real deployments.

Based on the above review, the following research gaps remain insufficiently addressed:

- Insufficient crack-specific geometric representation. Existing lightweight detectors and generic attention modules are mostly adapted from general-purpose visual detection frameworks and still insufficiently encode the slenderness, anisotropy, and directional continuity of cracks, especially for oblique, curved, intersecting, or discontinuous patterns.
- Limited detail preservation during multi-scale feature fusion. Existing multi-scale fusion strategies improve semantic aggregation, but high-frequency crack boundaries may still be attenuated during top-down upsampling, making subtle cracks difficult to localize under lightweight constraints.
- Weak geometry-aware regression supervision for slender targets. Existing IoU-based losses improve convergence and sample reweighting to some extent, but they provide limited supervision for short-side deviations and aspect ratio consistency in slender crack boxes, and their optimization pressure is not explicitly adapted to the quality regions where boundary refinement may be beneficial.
- Insufficient closed-loop validation from model design to edge deployment. Many studies report offline benchmark performance or theoretical efficiency, whereas fewer works validate the complete workflow from crack-aware module design to TensorRT acceleration and real robotic inspection.

To address these limitations, this work aims to provide a complete closed-loop workflow spanning geometry analysis, algorithm design, robust experimental validation, and engineering deployment. Our main contributions are as follows:

1. A crack-aware cross-dimensional fusion attention (CFCA) module that jointly models channel, spatial, and directional information to enhance anisotropic feature representation.
2. A dual-path feature enhancement module (DFEM) that decouples semantic refinement and edge enhancement to preserve high-frequency crack boundaries during upsampling.
3. A crack-aware localization-loss adaptation is designed for stable regression on slender targets. It combines an empirical smooth quality window adjustment with shape consistency constraints while preserving the original YOLO localization objective and calibrating CIoU-based supervision around selected quality boundaries.
4. End-to-end validation on a Jetson Orin NX platform with PyTorch 2.5.1 → ONNX 1.17.0 → TensorRT 8.5.0.2 deployment and real-world robotic inspection.

The remainder of the paper is organized as follows. Section 2 describes the proposed method in detail. Section 3 presents experimental results and an analysis. Section 4 discusses limitations and applicability. Section 5 concludes the paper and outlines future directions.

## 2. Method

### 2.1. Overall Framework

YOLO-Crack is built upon YOLOv11n and is designed for crack detection in edge-based mobile inspection. The central challenge in this setting lies in the mismatch between crack geometry and lightweight detection pipelines: cracks are typically slender, anisotropic, and weak in boundary contrast, while edge deployment requires efficient inference under limited resources. To address this problem, YOLO-Crack introduces three coordinated components into the baseline detector.

**Figure 1:**
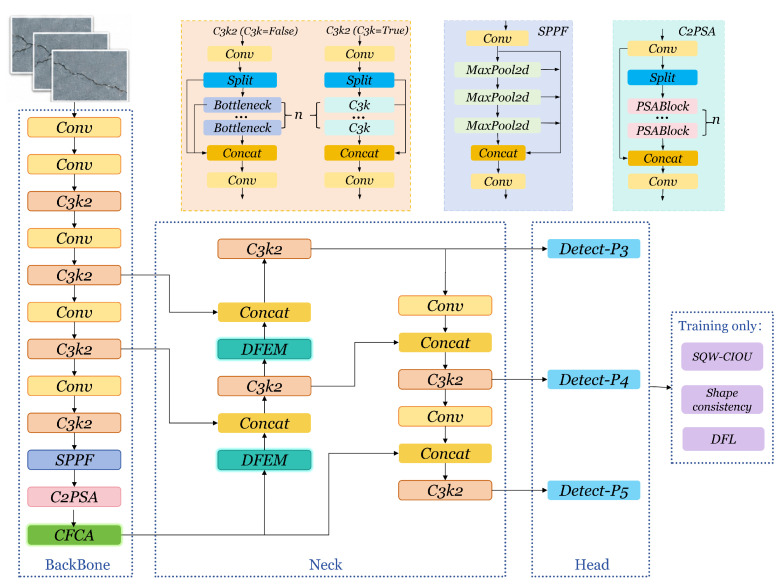
Overview of the YOLO-Crack framework.

As shown in Figure 1, a crack-aware cross-dimensional fusion attention (CFCA) module is inserted after the deep backbone stage to enhance crack-aware feature representation before neck propagation. A dual-path feature enhancement module (DFEM) is embedded into the top-down fusion pathway to preserve weak crack boundaries during multi-scale feature recovery. In addition, an empirical smooth quality window localization adjustment is applied during training to improve regression supervision for slender targets. These three components act on representation, feature fusion, and regression supervision, respectively, forming a unified geometry-guided framework for lightweight crack detection.

### 2.2. Crack-Aware Cross-Dimensional Fusion Attention

The objective of CFCA is to make the detector explicitly sensitive to crack anisotropy rather than merely increasing feature attention in a generic manner. In crack detection, the main difficulty is not that cracks are semantically complex objects, but that their discriminative cues are distributed across several weak and heterogeneous dimensions. Specifically, crack-background discrimination depends simultaneously on texture-sensitive channel responses, continuity-sensitive spatial localization, and orientation-sensitive elongated patterns. Generic attention designs can improve feature saliency, but they do not explicitly organize these crack-relevant cues according to the geometric characteristics of line-like targets.

CFCA is therefore designed as a crack-oriented anisotropic representation module. Instead of applying a standard attention block to globally reweight features, it explicitly decomposes crack representation into three complementary dimensions: channel semantics, spatial saliency, and directional awareness. These dimensions are modeled in parallel. This design is motivated by the observation that crack-related cues should be preserved and enhanced cooperatively rather than compressed through a sequential transformation. The parallel formulation enables the network to jointly balance texture discrimination, structural continuity, and directional enhancement.

#### Architecture and Implementation Details

**Figure 2:**
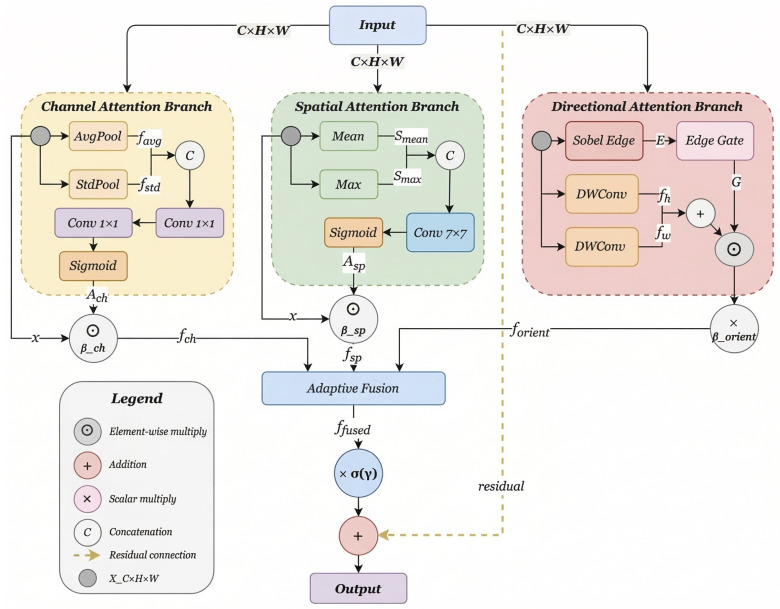
Architecture of the CFCA module.

As shown in Figure 2, given an input feature map $X\in R^{C\times H\times W}$, CFCA performs feature modulation along the channel, spatial, and directional dimensions through three parallel branches.

(1) Channel branch

The channel branch aims to distinguish crack texture from background texture at the semantic–statistical level. A channel descriptor based only on global average pooling may be insufficient when cracks and background regions have similar mean responses but differ in texture variability. To address this limitation, CFCA introduces a dual-statistics descriptor composed of average pooling and standard deviation pooling: $f_{\mathrm{avg}}=\mathrm{GAP}\left(X\right)$ and $f_{\mathrm{std}}=\mathrm{StdPool}\left(X\right)$. The former captures the mean channel response, whereas the latter provides complementary information about spatial response variability.

The two descriptors are concatenated and passed through a lightweight bottleneck multilayer perceptron to obtain the channel attention map:

(1) $$A_{\mathrm{ch}}=\sigma_{\mathrm{sig}}\left({\mathrm{FC}}_{2}\left(\mathrm{SiLU}\left({\mathrm{FC}}_{1}\left([f_{\mathrm{avg}},f_{\mathrm{std}}]\right)\right)\right)\right)\in R^{C}.$$

To enable both positive and negative residual modulation, the attention map is centered around zero:

(2) $$f_{\mathrm{ch}}=\sigma_{\mathrm{sig}}\left(\beta_{\mathrm{ch}}\right)\cdot \left(2A_{\mathrm{ch}}-1\right)⊙X.$$

Here, $\beta_{\mathrm{ch}}$ is a learnable scalar initialized to zero. The Sigmoid transformation therefore gives an initial gain of 0.5. Mapping $A_{\mathrm{ch}}$ from [0,1] to [−1,1] allows the branch to produce a residual modulation that can enhance crack-sensitive channel responses or suppress irrelevant responses.

(2) Spatial branch

The spatial branch is designed to highlight crack regions while preserving local structural continuity. It first computes channel-wise average pooling and max pooling:

(3) $$s_{\mathrm{token}}=\left[{\mathrm{Mean}}_{C}\left(X\right),{\mathrm{Max}}_{C}\left(X\right)\right]\in R^{2\times H\times W}.$$

The average-pooled map provides contextual information about the overall spatial structure, whereas the max-pooled map emphasizes locally strong responses, such as thin crack edges. The concatenated token is processed by a 7×7 convolution to generate the spatial attention map:

(4) $$A_{\mathrm{sp}}=\sigma_{\mathrm{sig}}\left(\mathrm{BN}\left(\phi_{7\times 7}\left(s_{\mathrm{token}}\right)\right)\right).$$

The corresponding spatial residual is defined as

(5) $$f_{\mathrm{sp}}=\sigma_{\mathrm{sig}}\left(\beta_{\mathrm{sp}}\right)\cdot \left(2A_{\mathrm{sp}}-1\right)⊙X,$$

where $\beta_{\mathrm{sp}}$ is a learnable scalar initialized to zero, corresponding to an initial Sigmoid gain of 0.5. This branch produces a spatial residual that enhances crack-relevant regions and suppresses responses associated with background interference.

(3) Direction branch

The directional branch is designed to enhance elongated structural responses associated with crack geometry. Instead of using an isotropic convolution, the branch employs axial depthwise convolutions to encode strip-like context along two canonical directions [45]:

(6) $$\begin{array}{ccc} f_{h} & = & \mathrm{SiLU}\left(\mathrm{BN}\left(\phi_{7\times 1}\left(X\right)\right)\right),\;f_{w}=\mathrm{SiLU}\left(\mathrm{BN}\left(\phi_{1\times 7}\left(X\right)\right)\right),\\ f_{\mathrm{orient}} & = & \frac{1}{2}\left(f_{h}+f_{w}\right)\in R^{C\times H\times W}. \end{array}$$

The two axial operators do not assume that cracks are restricted to horizontal or vertical orientations. Instead, they provide complementary directional bases for elongated structures. Their combined responses can also support the representation of oblique and mildly curved cracks when integrated with the spatial branch.

Directional filters may also respond to structured background textures. The Sobel prior supplies low-level boundary cues, while the learnable projection determines whether these cues should enhance or suppress directional responses:

(7) $$E=\mathrm{SobelEdge}\left(X\right)\in R^{1\times H\times W}.$$

In the implementation, the Sobel operator is initialized with standard Sobel kernels and remains non-trainable. It therefore introduces no additional learnable parameters. The Sobel response is not used as a hard mask or an independent supervision signal, but serves only as an auxiliary edge prior for soft gating.

The one-channel edge prior is projected into a channel-wise gating map through a learnable 3×3 convolution followed by batch normalization and a Sigmoid activation:

(8) $$G=\sigma_{\mathrm{sig}}\left(\mathrm{BN}\left({\mathrm{Conv}}_{3\times 3}\left(E\right)\right)\right)\in R^{C\times H\times W}.$$

The learnable projection adapts the fixed Sobel prior to the feature representation and reduces the influence of isolated edge responses. The directional residual is then computed as

(9) $$f_{\mathrm{dir}}=\sigma_{\mathrm{sig}}\left(\beta_{\mathrm{orient}}\right)\cdot G⊙\left(f_{\mathrm{orient}}-X\right),$$

where $\beta_{\mathrm{orient}}$ is a learnable scalar initialized to zero, corresponding to an initial Sigmoid gain of 0.5.

This residual formulation prevents the directional branch from directly replacing the original feature representation. Instead, it injects a gated directional residual whose magnitude is modulated by the learned edge gate. Although the Sobel kernels remain fixed, the axial convolutions, the 3×3 gate projection, the gain parameter, and the subsequent fusion parameters are jointly optimized during training.

(4) Adaptive multi-dimensional fusion

The contributions of the three residual branches are balanced using learnable fusion parameters $\alpha =[\alpha_{0},\alpha_{1},\alpha_{2}]$, which are normalized by a Softmax function. A learnable global residual scaling parameter γ is initialized to −1:

(10) $$\begin{array}{ccc} w & = & \mathrm{Softmax}\left(\alpha \right),\;f_{\mathrm{fused}}=w_{0}f_{\mathrm{dir}}+w_{1}f_{\mathrm{ch}}+w_{2}f_{\mathrm{sp}}. \end{array}$$

The final CFCA output is defined as

(11) $$X_{\mathrm{out}}=X+\sigma_{\mathrm{sig}}\left(\gamma \right)\cdot f_{\mathrm{fused}}.$$

Initializing γ=−1 gives $\sigma_{\mathrm{sig}}\left(\gamma \right)\approx 0.27$, which limits the magnitude of the fused residual during the early stage of training. The fusion weights are jointly optimized to balance the overall contributions of the directional, channel, and spatial residuals.

### 2.3. Dual-Path Feature Enhancement Module

In lightweight detectors, top-down multi-scale fusion is essential for recovering spatial details from deep semantic features. However, for crack detection, the limitation of top-down fusion should not be regarded solely as a semantic multi-scale problem. For thin and low-contrast cracks, the same upsampling operation is required to propagate high-level semantics while preserving weak boundary details. These two objectives are not fully aligned. Semantic propagation favors smooth and stable responses, whereas boundary preservation depends on local transitions that can be attenuated during repeated interpolation.

DFEM is designed to address these two objectives through a lightweight dual-path architecture. Rather than replacing bilinear interpolation with a computationally intensive upsampling operator, DFEM employs separate but coordinated branches for feature refinement and boundary-response enhancement. This design is motivated by the observation that subtle crack responses may be weakened during top-down feature propagation, particularly when their localization depends on narrow and low-contrast boundaries.

**Figure 3:**
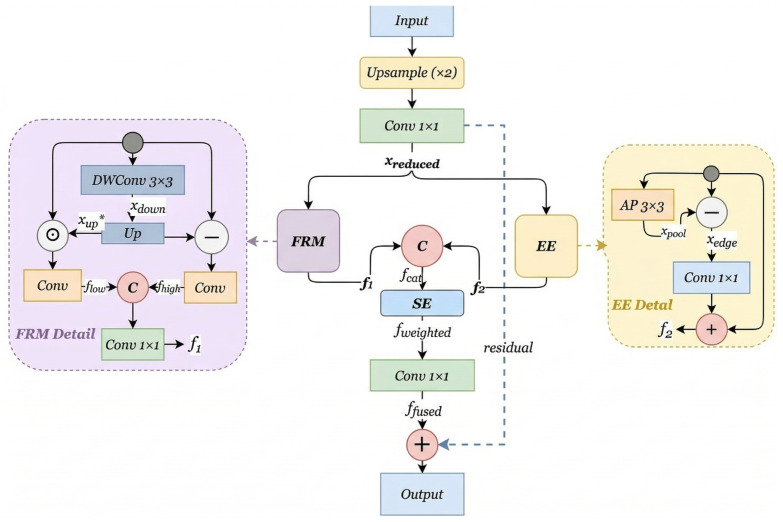
Architecture of the DFEM module.

As illustrated in Figure 3, given an input feature $X_{\mathrm{in}}\in R^{C_{\mathrm{in}}\times H\times W}$, DFEM first upsamples it to 2H×2W using bilinear interpolation: $x_{\mathrm{up}}=\mathrm{Interpolate}\left(X_{\mathrm{in}}\right).$ The channel dimension is then reduced to $C^{\prime }=C_{\mathrm{in}}/4$ through a 1×1 convolution:

(12) $$x_{\mathrm{reduced}}=\mathrm{SiLU}\left(\mathrm{BN}\left(\phi_{1\times 1}\left(x_{\mathrm{up}}\right)\right)\right).$$

The reduced feature is processed by two parallel branches. The Feature Refinement Module (FRM) constructs complementary responses through a downsampling and interpolation round trip, whereas the Edge Enhancer (EE) extracts local residual responses based on a high-pass filtering principle. The two outputs are concatenated, recalibrated using an SE module, and fused through a convolutional projection.

#### 2.3.1. Feature Refinement Module

The FRM branch is designed to refine structurally stable responses while identifying feature components that are sensitive to resolution changes. The reduced feature is first downsampled and then interpolated back to its original spatial resolution:

(13) $$\begin{array}{c} x_{\mathrm{down}}=\phi_{\mathrm{down}}\left(x_{\mathrm{reduced}}\right),\\ {\tilde{x}}_{\mathrm{up}}=\mathrm{Interpolate}\left(x_{\mathrm{down}}\right). \end{array}$$

Here, ${\tilde{x}}_{\mathrm{up}}$ denotes the reconstructed feature after the resolution round trip and has the same spatial dimensions as $x_{\mathrm{reduced}}$. Based on these two features, FRM constructs a multiplicative response and a difference response.

The multiplicative response is defined as $x_{\mathrm{reduced}}⊙{\tilde{x}}_{\mathrm{up}}$. It emphasizes responses that remain consistent after downsampling and interpolation. The difference response, $x_{\mathrm{reduced}}-{\tilde{x}}_{\mathrm{up}}$, highlights feature components attenuated by the resolution round trip. The two responses are processed independently and then fused:

(14) $$\begin{array}{ccc} f_{\mathrm{low}} & = & \mathrm{Norm}\left(\phi_{\mathrm{lconv}}\left(\mathrm{GELU}\left(x_{\mathrm{reduced}}⊙{\tilde{x}}_{\mathrm{up}}\right)\right)\right),\\ f_{\mathrm{high}} & = & \mathrm{Norm}\left(\phi_{\mathrm{hconv}}\left(\mathrm{GELU}\left(x_{\mathrm{reduced}}-{\tilde{x}}_{\mathrm{up}}\right)\right)\right),\\ f_{1} & = & \mathrm{GELU}\left(\phi_{\mathrm{proj}}\left([f_{\mathrm{low}},f_{\mathrm{high}}]\right)\right). \end{array}$$

Although the two responses are denoted by $f_{\mathrm{low}}$ and $f_{\mathrm{high}}$ for consistency with the module architecture, they do not constitute a strict frequency-domain decomposition. Instead, they represent information preserved during the resolution round trip and information weakened by that process, respectively.

The role of FRM is therefore not to reconstruct edges through an explicit frequency-domain operation. It uses a lightweight resolution round-trip to jointly model structurally stable responses and smoothing-sensitive residuals, thereby retaining crack-relevant information with limited computational overhead.

#### 2.3.2. Edge Enhancer

While FRM captures complementary responses associated with resolution changes, the EE branch provides a dedicated path for local boundary enhancement. A locally averaged feature is first obtained using a 3×3 average-pooling operation:

(15) $$x_{\mathrm{pool}}={\mathrm{AvgPool}}_{3\times 3}\left(x_{\mathrm{reduced}}\right).$$

The local average is then subtracted from the reduced feature to produce an edge-sensitive residual:

(16) $$x_{\mathrm{edge}}=x_{\mathrm{reduced}}-x_{\mathrm{pool}}.$$

The residual is projected through a 1×1 convolution, batch normalization, and a Sigmoid activation. The resulting bounded enhancement term is added to the reduced feature:

(17) $$f_{2}=x_{\mathrm{reduced}}+\sigma_{\mathrm{sig}}\left(\mathrm{BN}\left(\phi_{1\times 1}^{\prime }\left(x_{\mathrm{edge}}\right)\right)\right).$$

This branch enhances local responses associated with weak crack boundaries. The residual connection preserves the reduced feature representation, whereas the Sigmoid activation bounds the magnitude of the added enhancement term. This formulation limits uncontrolled amplification, although edge-like background textures may still produce responses and must subsequently be suppressed through joint feature fusion.

#### 2.3.3. SE-Based Feature Fusion and Residual Connection

The outputs of FRM and EE provide complementary information. FRM models responses associated with resolution consistency and smoothing sensitivity, whereas EE emphasizes local boundary residuals. The two branch outputs are first concatenated: $f_{\mathrm{cat}}=\left[f_{1},f_{2}\right].$ An SE module is then applied to recalibrate the concatenated representation at the channel level. A global channel descriptor is obtained through global average pooling: $z=\mathrm{GAP}\left(f_{\mathrm{cat}}\right).$

The channel weights are generated through a two-layer multilayer perceptron:

(18) $$w=\sigma_{\mathrm{sig}}\left({\mathrm{FC}}_{2}\left(\mathrm{SiLU}\left({\mathrm{FC}}_{1}\left(z\right)\right)\right)\right).$$

The recalibrated feature and the subsequent fused representation are defined as

(19) $$\begin{array}{ccc} f_{\mathrm{weighted}} & = & f_{\mathrm{cat}}⊙w,\\ f_{\mathrm{fused}} & = & \mathrm{SiLU}\left(\mathrm{BN}\left(\phi_{\mathrm{fuse}}\left(f_{\mathrm{weighted}}\right)\right)\right). \end{array}$$

The SE module assigns channel-wise weights to the joint FRM and EE representation rather than producing an explicit scalar weight for each branch. It therefore performs channel recalibration after branch concatenation.

Finally, the DFEM output is obtained through a residual connection:

(20) $$\mathrm{out}=f_{\mathrm{fused}}+\phi_{\mathrm{res}}\left(x_{\mathrm{up}}\right),$$

where $\phi_{\mathrm{res}}$ aligns the channel dimension of the initially upsampled feature, typically using a 1×1 projection or an identity mapping when the input and output channel dimensions are equal.

### 2.4. Crack-Aware Localization-Loss Adaptation

Enhancing representation and fusion alone is not sufficient for crack detection, because the supervision signal must also be aligned with the geometry of the target. For slender crack boxes, localization errors are often dominated less by coarse overlap and more by short-side deviation and aspect ratio inconsistency. A prediction may overlap well with the ground truth along the long axis while still being inaccurate in width or boundary placement. Under this condition, conventional IoU-dominated regression losses provide limited guidance for the type of refinement that matters most in crack detection.

The proposed localization adjustment is an empirical extension of the original YOLO regression objective rather than a replacement for it. It keeps CIoU as the dominant localization signal and adds two complementary terms: a smooth quality window CIoU adjustment that calibrates the loss profile around selected quality boundaries, and a shape consistency regularizer that explicitly supervises the scale and aspect ratio of slender targets. This design is intended to provide a soft, differentiable alternative to hard sample partitioning while retaining the stable behavior of the baseline detector.

#### 2.4.1. Smooth Quality Window CIoU

Let *u* denote the CIoU quality score between a predicted box and its assigned target. The key idea is to treat localization quality as a continuous state rather than dividing positive samples into fixed hard/easy groups. To this end, we introduce a smooth quality window mapping:

(21) $$M_{\mathrm{SQW}}\left(u\right)=\mathrm{clip}\left[\frac{\sigma \;\left(\frac{u-t_{l}}{\tau }\right)-\sigma \;\left(\frac{u-t_{h}}{\tau }\right)}{\sigma \;\left(\frac{1-t_{l}}{\tau }\right)-\sigma \;\left(\frac{-t_{h}}{\tau }\right)},0,1\right],$$

where $t_{l}$ and $t_{h}$ define the lower and upper quality boundaries, respectively, and τ controls the transition smoothness. In the reported implementation, these hyperparameters are set empirically as $t_{l}=0.3$, $t_{h}=0.70$, and τ=0.10. The clipping operation ensures numerical stability because CIoU scores may be negative for poorly aligned boxes.

Based on this mapping, the smooth quality window CIoU loss is defined as

(22) $$L_{\mathrm{SQW}-\mathrm{CIoU}}=\left(1-\lambda \right)\left(1-u\right)+\lambda \left[1-M_{\mathrm{SQW}}\left(u\right)\right].$$

Different from conventional sample reweighting, the proposed formulation does not multiply the CIoU loss by an independent sample weight. Instead, it linearly combines the original CIoU loss with a smooth window adjustment term. The original CIoU component preserves the primary localization supervision, whereas the window term empirically reshapes the loss and gradient profiles around the selected quality boundaries. In this sense, the term acts as a bounded quality calibration mechanism: it changes how regression pressure is distributed over the CIoU range without altering the sample assignment strategy or introducing a discontinuous partition of positive samples. This formulation is consistent with the implementation in BboxLoss.

#### 2.4.2. Shape Consistency Constraint

Let $\left(w_{p},h_{p}\right)$ and $\left(w_{g},h_{g}\right)$ denote the dimensions of the predicted and target boxes, respectively. The scale and aspect ratio discrepancies are defined as

(23) $$\begin{array}{cc} L_{\mathrm{scale}}\; & =\frac{1}{2}\left(\frac{|w_{p}-w_{g}|}{w_{g}+\epsilon }+\frac{|h_{p}-h_{g}|}{h_{g}+\epsilon }\right),\\ L_{\mathrm{aspect}}\; & =\mathrm{SmoothL1}\left(\log\frac{w_{p}}{h_{p}}-\log\frac{w_{g}}{h_{g}}\right). \end{array}$$

We define target slenderness as $\rho_{g}=\min\left(w_{g},h_{g}\right)/\max\left(w_{g},h_{g}\right)$ and use

(24) $$\omega \left(\rho_{g}\right)=\left\{\begin{array}{cc} 2.0, & \rho_{g}<0.6,\\ 1.0, & \mathrm{otherwise}. \end{array}\right.$$

The shape consistency loss is therefore

(25) $$L_{\mathrm{shape}}=\omega \left(\rho_{g}\right)\left(L_{\mathrm{scale}}+L_{\mathrm{aspect}}\right).$$

For extremely slender targets, the aspect ratio discrepancy is further reinforced as

(26) $$L_{\mathrm{ext}-\mathrm{ar}}=I\left(\rho_{g}<0.4\right)\;L_{\mathrm{aspect}}.$$

#### 2.4.3. Overall Training Objective

The localization term is

(27) $$L_{\mathrm{loc}}=L_{\mathrm{SQW}-\mathrm{CIoU}}+0.20\;L_{\mathrm{shape}}+0.15\;L_{\mathrm{ext}-\mathrm{ar}}.$$

The complete detection objective is

(28) $$L_{\mathrm{total}}=\lambda_{\mathrm{box}}L_{\mathrm{loc}}+\lambda_{\mathrm{cls}}L_{\mathrm{cls}}+\lambda_{\mathrm{dfl}}L_{\mathrm{DFL}}.$$

All per-sample localization terms are weighted by the assigned target scores and normalized by the sum of positive target scores, following the original YOLO training pipeline. In the reported experiments, the center-alignment regularizer is disabled by setting its weight to zero.

## 3. Experiments and Results

We evaluate the proposed YOLO-Crack framework from six perspectives: (i) effectiveness on the main Crack500 benchmark, (ii) contribution of each proposed component, (iii) statistical reliability under different random splits, (iv) cross-dataset generalization, (v) robustness under challenging visual conditions, and (vi) edge-device deployment evaluation.

### 3.1. Experimental Setup

#### 3.1.1. Datasets

We evaluate YOLO-Crack on the Crack500 benchmark dataset. Crack500 contains 500 crack images with a resolution of 2000×1500 pixels, covering diverse infrastructure scenes such as concrete and asphalt pavements. After cropping, there are a total of 3368 640×360 images. The dataset exhibits substantial variations in illumination and complex backgrounds, and cracks often appear with low contrast and intermittent visibility, making them likely to be confused with background texture edges, which can lead to false positives and missed detections.

The original Crack500 dataset provides only pixel-level segmentation masks, but lacks the bounding-box annotations required by YOLO-style detectors. In early explorations, we also attempted finer-grained segment-wise box annotation; however, we found that overly dense small-box labeling often causes a clear performance degradation. We attribute this to the fact that excessive emphasis on local fragments weakens the model’s ability to learn the global continuity of cracks and to generalize.

Based on this observation, we adopt a more robust policy that allows a bounding box to include a moderate amount of background to maintain crack integrity, which better matches the long-range nature of cracks. Specifically, we keep the bounding boxes generated by a contour-based algorithm with limited background tolerance to preserve crack completeness and only remove obvious annotation noise to maximize data diversity. The final dataset split consists of 1896 training images, 348 validation images, and 1124 test images.

**Figure 4:**
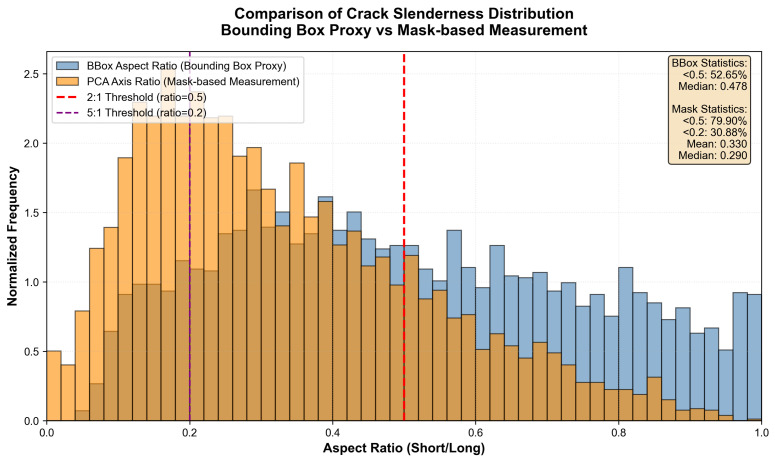
Distribution of crack slenderness estimated by bounding boxes (blue) and PCA-based masks (orange).

After annotation, we observe that crack instances exhibit a typical slender geometry and diverse orientations. Based on statistics of 4120 annotated boxes (a bbox proxy), 52.6% of samples satisfy min(w,h)/max(w,h)<0.5, with a median of 0.478 (approximately 1:2.1) and an extreme value of about 0.046 (approximately 1:21.7). However, box-based measurement is subject to systematic bias. To preserve crack integrity, the bounding box often includes extra background and thus produces conservative shape statistics. Figure 4 shows the comparison of slenderness estimated by bounding boxes and PCA-based masks. On 7972 crack instances from 1896 images, the mean ratio is 0.330, the median is 0.290, 79.90% of instances are smaller than 0.5, and 30.88% are smaller than 0.2. Mask-based measurement reveals substantially stronger slenderness: the median aspect ratio changes from 1:2.1 (box proxy) to 1:3.5, highlighting the extreme geometry of cracks. This quantitative evidence motivates the direction-aware design of CFCA and the detail-preserving strategy of DFEM.

To further evaluate cross-dataset generalization, we additionally use Crack-Seg, DeepCrack, and CrackTree200 as external test datasets. These datasets differ from Crack500 in acquisition conditions, surface materials, crack density, annotation granularity, and background complexity. For consistency with the detection paradigm, segmentation masks or crack regions in these datasets are converted into bounding-box labels using the same contour-based conversion protocol as Crack500. Since the objective of this part is to evaluate transferability from Crack500 to unseen data distributions, the models are trained on Crack500 and directly evaluated on the external datasets without additional fine-tuning. The dataset details are summarized in Table 1.

#### 3.1.2. Implementation Details

All models are implemented using PyTorch and the Ultralytics YOLO framework. Unless otherwise specified, the input image size is set to 640×640, the batch size is 16, and all models are trained for 100 epochs. The AdamW optimizer is adopted with an initial learning rate of 0.001 and a weight decay of 0.0005. Standard data augmentation strategies, including random scaling, flipping, mosaic augmentation, and color jittering, are used during training.

The proposed YOLO-Crack is built upon YOLOv11n. To ensure fair comparison, all baseline models and ablation variants are trained using the same training protocol, input resolution, and data split. For deployment evaluation, trained PyTorch models are exported to ONNX and then converted to TensorRT engines on the Jetson Orin NX platform.

#### 3.1.3. Evaluation Metrics

We report precision, recall, and AP at an IoU threshold of 0.5 (mAP@0.5) using the Ultralytics evaluation pipeline. Model complexity is evaluated using parameter count and GFLOPs. Inference performance is evaluated using latency and frames per second (FPS). For edge-device experiments, we report both pure TensorRT inference speed and end-to-end throughput in the ROS-based robotic pipeline.

### 3.2. Ablation Studies

#### 3.2.1. Ablation on Individual Modules

To validate the effectiveness of each component, we conduct ablation studies using YOLOv11n as the baseline. Table 2 reports the individual contributions of CFCA, DFEM, and the smooth quality window localization adjustment, as well as their combined effect.

**Figure 5:**
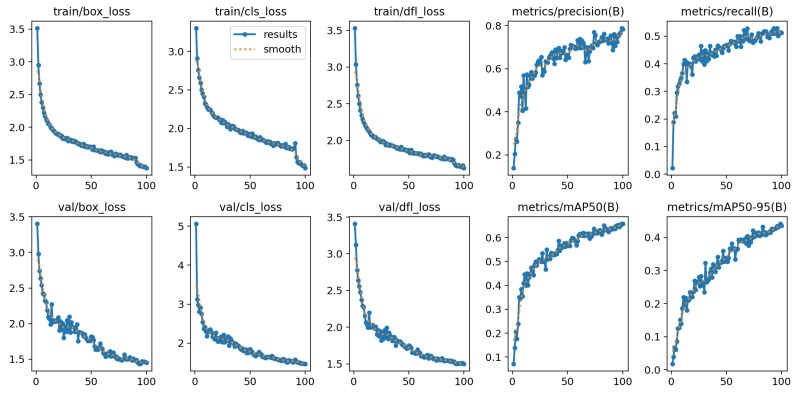
Training convergence curves of YOLO-Crack.

Figure 5 shows the training dynamics. Loss curves indicate stable convergence without obvious overfitting, while mAP@0.5 and mAP@0.5:0.95 steadily increase and stabilize at around 0.65 and 0.45, respectively.

#### 3.2.2. Design Space Exploration of DFEM

To identify the optimal branch configuration in DFEM, we perform a systematic ablation using SE fusion for fair comparison. Table 3 reveals two key findings: (1) using only FRM or only EE yields a clear weakness in the precision–recall trade-off, confirming that explicit decoupling and synergy of semantic and edge cues are necessary; and (2) a three-branch design (FRM + EE + SCM [46]) degrades performance due to feature interference. The two-branch structure achieves the best balance.

#### 3.2.3. Orientation Sensitivity of CFCA

To investigate whether the use of 1 × 7 and 7 × 1 axial depth convolution in the CFCA direction branch is only suitable for horizontal/vertical cracks and cannot adapt to oblique cracks, the PCA algorithm is used to estimate the principal axis direction of the crack mask. The samples are categorized into near-horizontal, near-vertical, and diagonal groups according to the dominant orientation. We compare YOLO-Crack without the direction branch and full YOLO-Crack. The results are shown in Table 4.

The full CFCA achieves a higher mAP@0.5 across all three orientation groups. The improvement is most evident for horizontal cracks, where the mAP@0.5 increases from 64.3% to 66.4%, and the precision increases from 79.5% to 84.2%. For vertical cracks, the precision increases substantially from 52.5% to 65.5%, although recall slightly decreases, indicating that the direction branch suppresses some ambiguous vertical background textures while improving prediction reliability. For diagonal cracks, the full CFCA still improves mAP@0.5 from 66.3% to 67.0% and recall from 50.3% to 52.4%. These results indicate that the direction branch does not simply assume cracks to be horizontal or vertical. Instead, the axial convolutions provide lightweight elongated directional bases, while oblique crack structures are further handled through spatial saliency, Sobel-guided edge gating, and adaptive multi-dimensional fusion. Nevertheless, this analysis only evaluates dominant local orientation. It does not imply that a bounding-box detector can fully model complex crack topology such as dense branching, reticulation, or highly tortuous crack networks; these cases are further examined in the cross-dataset evaluation.

### 3.3. Comparison with Lightweight YOLO Detectors

To evaluate the effectiveness of YOLO-Crack under a controlled detection setting, we compare it with representative lightweight YOLO detectors. All models are retrained on the processed Crack500 detection annotations using the same input size, optimizer, training schedule, and data split. The results are shown in Table 5.

YOLO-Crack achieves the best overall performance among the compared YOLO baselines. Compared with YOLOv11n, YOLO-Crack improves mAP@0.5 by 2.9 percentage points while increasing parameters by only 0.15 M. These results indicate that the performance gain of YOLO-Crack comes not from simply enlarging the model, but from crack-specific representation, detail-preserving fusion, and empirically calibrated geometry-aware regression supervision.

Direct comparison with many recent crack-specific methods, including highly accurate segmentation-oriented or edge-deployable models, is not straightforward because the task definitions, annotation formats, datasets, and hardware settings differ. Segmentation methods usually report pixel-level metrics such as IoU or F1, whereas this work reports bounding-box detection metrics after converting crack masks to detection boxes. Therefore, Table 5 conducts comparisons among lightweight YOLO models under identical experimental settings to verify whether our crack-aware modules can boost the performance of lightweight detectors for practical deployment.

**Figure 6:**
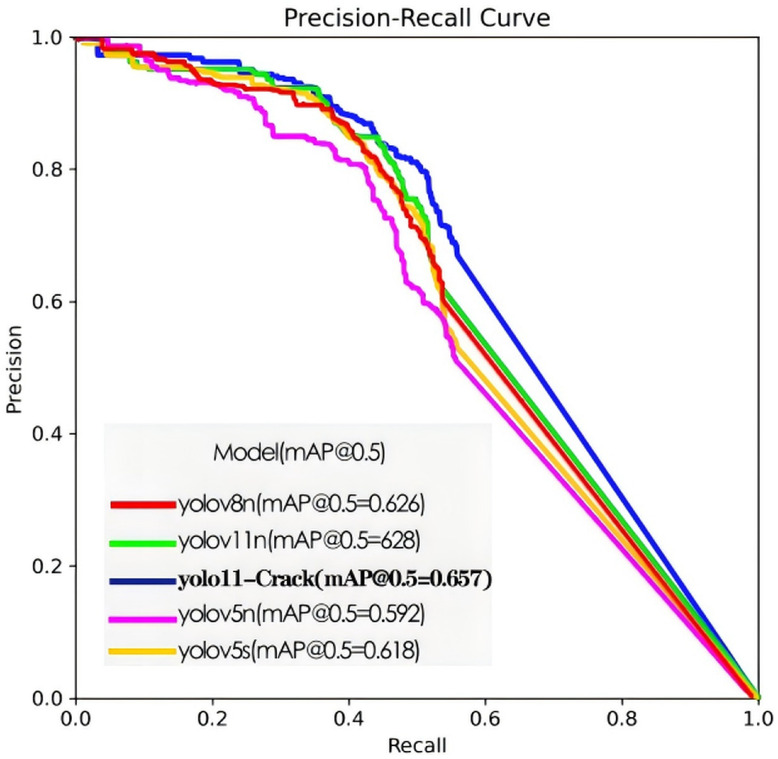
Precision–recall curves.

Figure 6 shows the comparison of precision–recall curves, where the YOLO-Crack curve (blue) lies above the baseline over most of the evaluated recall range.

### 3.4. Statistical Reliability Under Multiple Random Splits

To reduce the dependence of the reported results on a single dataset partition, we generated three independent train, validation, and test splits of the complete Crack500-derived detection dataset using random seeds of 2023, 2024, and 2025. For each split, all 3368 images were randomly shuffled and divided using the same proportions as the main experiment: 1896 images for training, 348 images for validation, and 1124 images for testing. Although the subset sizes remained unchanged, the image composition of the training, validation, and test sets differed across the three splits.

For every split, YOLOv11n and YOLO-Crack were trained from scratch using the same optimization settings and evaluated on the corresponding evaluation subset. This protocol was used to assess whether the observed performance improvement was robust to changes in dataset partitioning.

As shown in Table 6, the lightweight YOLOv11n exhibits substantial performance variation under different dataset splits, with a standard deviation of mAP@0.5 of ±2.9%. YOLO-Crack exhibits lower variability across the three evaluated splits, with a standard deviation of ±1.9%, and consistently higher mAP@0.5 than the baseline, with an average improvement of 2.1 percentage points. Because the data partitions are changed in this experiment, the variance reflects both split difficulty and training randomness rather than training stability alone.

### 3.5. Qualitative Results

**Figure 7:**
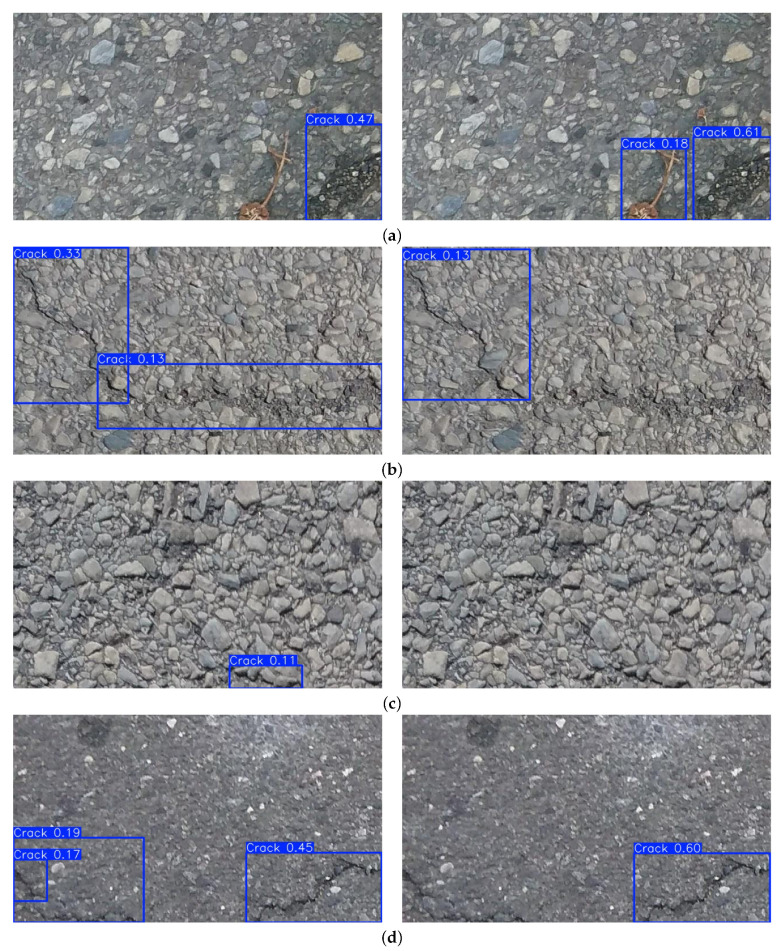
Qualitative comparison between YOLOv11n and YOLO-Crack. (**a**) Confidence improvement; (**b**) detection of subtle cracks; (**c**) robustness under complex backgrounds; (**d**) multiple cracks and complex shapes.

Figure 7 visualizes detection results of the YOLOv11n baseline and YOLO-Crack in representative scenarios.

As shown in Figure 7a, YOLO-Crack consistently produces higher confidence and better-fitting bounding boxes than the baseline. For subtle cracks (b), the baseline generates only low-confidence responses, whereas YOLO-Crack provides more continuous coverage. Under complex background interference (c), YOLO-Crack more reliably distinguishes cracks from background clutter. For multiple cracks with complex shapes (d), YOLO-Crack achieves more complete detection, while the baseline often misses targets or produces incomplete predictions.

### 3.6. Cross-Dataset Generalization

To further evaluate the generalization ability of the model outside the training distribution, this paper adopts a direct cross-dataset testing method for supplementary validation.

As shown in Table 7, the overall detection performance obtained via direct cross-dataset evaluation is considerably inferior to the in-domain results tested on Crack500, which demonstrates remarkable distribution shifts across different crack datasets. Especially on the CrackTree200 benchmark, the mAP@0.5 of YOLOv11n is only 14.6%, while the proposed YOLO-Crack reaches 20.7%, yet the absolute detection accuracy remains unsatisfactory. This phenomenon mainly originates from discrepancies in crack morphology and annotation distribution of target datasets. Unlike Crack500, which is dominated by relatively continuous linear cracks, CrackTree200 and DeepCrack contain abundant bifurcated, reticulated, densely clustered, or highly tortuous cracks. These structures are difficult to describe with a small number of horizontal bounding boxes because a box may enclose large non-crack regions while failing to represent crack skeletons, branch points, and local curvature. In addition, these datasets were originally designed for semantic segmentation tasks; after the mask-to-bounding-box conversion, the generated annotations are susceptible to connected-component segmentation, bounding-box scale variation, and crack density, which pose challenges for detection evaluation.

Even so, YOLO-Crack consistently outperforms YOLOv11n across all three external test datasets. On Crack-Seg, our method improves mAP@0.5 from 40.1% to 46.8%; on CrackTree200, mAP@0.5 rises from 14.6% to 20.7%; on DeepCrack, precision is boosted from 45.8% to 51.4%, accompanied by a marginal improvement in recall. These results should be interpreted as evidence of relative improvement under domain shift rather than as evidence that the current detector fully solves cross-dataset crack perception.

### 3.7. Robustness Under Challenging Conditions

To further explore the model stability against common visual distortions encountered in practical pavement inspection, four types of image perturbations are artificially constructed on the Crack500 validation set, namely, Gaussian noise, low illumination, high illumination and crack-like background distractors. Specifically, Gaussian noise and illumination variation simulate degraded image acquisition quality and fluctuating ambient lighting; crack-like distractors are adopted to mimic shadow, slender debris and stains with linear textures analogous to real pavement cracks. The results are shown in Table 8.

Experimental results indicate that YOLO-Crack maintains a consistent advantage over YOLOv11n under Gaussian noise, low-light, and overexposure conditions. Under Gaussian noise, YOLO-Crack achieves an mAP@0.5 of 62.0%, 3.7 percentage points higher than YOLOv11n; under low illumination, mAP@0.5 increases from 58.3% to 61.7%; under high illumination, mAP@0.5 increases from 61.2% to 64.2%. These results suggest that the complete YOLO-Crack framework is more robust than the YOLOv11n baseline under the evaluated imaging degradations.

By contrast, crack-like distractors constitute the most challenging test case. Relative to the main clean Crack500 benchmark, the mAP@0.5 of YOLOv11n drops from 62.8% to 41.7%, and YOLO-Crack declines from 65.7% to 42.3%. Although YOLO-Crack still surpasses the baseline marginally, the performance margin shrinks compared with other disturbance cases. This reveals that bounding-box supervision based only on RGB inputs struggles to distinguish true cracks from non-crack linear textures with similar gradient characteristics. Because YOLO-Crack is designed to enhance slender crack features with distinct edges, crack-like distractors with strong linear gradients can still induce false positives.

**Figure 8:**
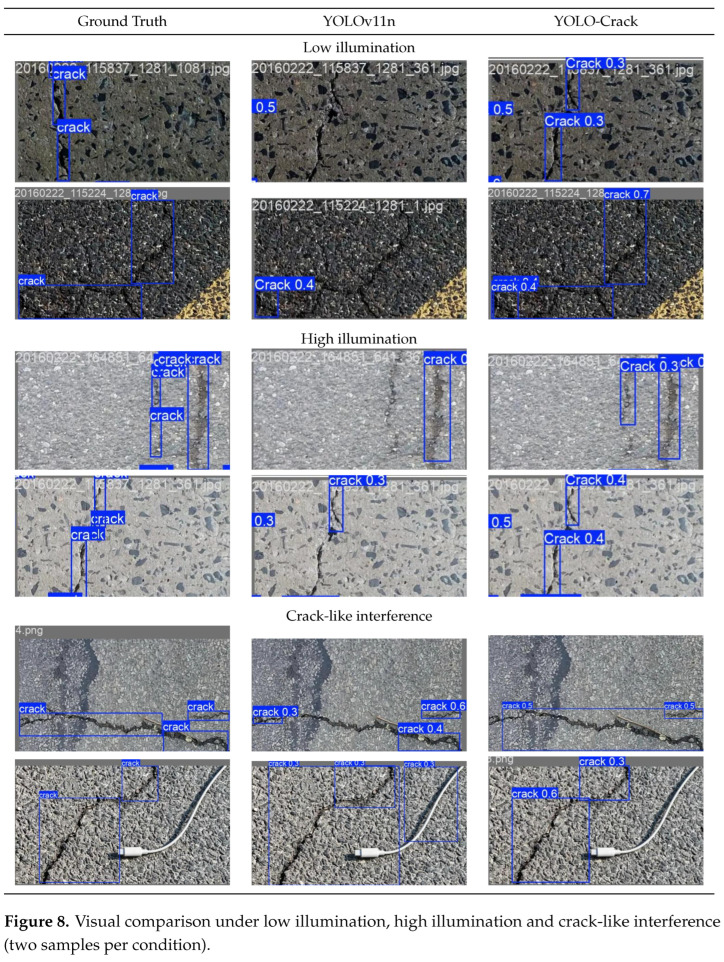
Visual comparison under low illumination, high illumination and crack-like interference (two samples per condition).

Figure 8 provides qualitative detection comparisons under three typical challenging environments. Compared with YOLOv11n, YOLO-Crack alleviates missed detections under varying illumination and reduces some false responses caused by crack-shaped background clutter, which is consistent with the quantitative robustness results listed in Table 8.

In summary, YOLO-Crack achieves steady comparative advantages under conventional image degradation but suffers prominent limitations against crack-shaped background interference. Such observation aligns with the conclusion drawn from cross-dataset tests: detection accuracy degrades severely once the foreground morphology or background distribution deviates drastically from the Crack500 training domain. Relevant discussions and potential solutions, including segmentation-guided supervision and RGB-D multimodal fusion for robust open-world detection, will be elaborated on in Section 4.

### 3.8. Edge-Device Deployment Evaluation

To validate the practicality of YOLO-Crack under resource constraints, we deployed it on an NVIDIA Jetson Orin NX edge platform (16 GB, 100 TOPS, JetPack 5.1.1) and evaluated the full conversion and inference pipeline.

#### 3.8.1. Model Conversion and TensorRT Acceleration

The trained PyTorch model (.pt) is first exported to ONNX, then converted to an optimized TensorRT engine. We generated both FP32 (full precision) and FP16 (half precision) engines. For each configuration, we ran 1000 inferences and reported the average latency (with 200 warmup runs, ignoring the top/bottom 5% outliers). The results are summarized in Table 9.

It should be clarified that this work uses FP32 and FP16 TensorRT inference only. No INT8 post-training quantization is performed. Therefore, no calibration dataset is required for the FP16 engine. Calibration is only required for INT8 quantization, where representative data are used to estimate activation ranges. In our implementation, the FP16 engine is generated by enabling half-precision inference during TensorRT engine building.

FP16 TensorRT inference provides substantial acceleration. YOLOv11n and YOLO-Crack achieve speedups of 1.75 times and 1.64 times, respectively. Although the additional overhead from CFCA and DFEM reduces FPS from 100.5 to 58.7, it creates a 2.9% mAP gain while still exceeding the real-time threshold of 25 FPS. The speed gap mainly stems from the multi-branch structure, which reduces the optimization space of TensorRT kernel fusion.

#### 3.8.2. Real-World Robotic Deployment

We integrated the optimized model into a ROS system and developed a dedicated crack detection node (crack_detector_node). The node subscribes to the /camera/color/image_raw topic published by an Orbbec Astra Pro Plus camera, performs TensorRT FP16 inference, and publishes detection outputs (bounding boxes, class, confidence) to /crack_detection/annotated_image for real-time monitoring. A synchronous callback is used to ensure that detection results strictly align with the camera timestamps.

Field tests are conducted on campus roads, where the robot autonomously cruises at 0.2 m/s with continuous detection. To quantify end-to-end performance, we compute the actual throughput during stable operation (after warmup). Across multiple 5 min trials, the system achieves an average throughput of 25.5 ± 1.2 (mean ± standard deviation over 500+ frames), meeting real-time video processing requirements (≥25 FPS).

Performance profiling indicates that the measured latency of the Ultralytics inference framework is about 28 ms, including Python 3.10.20-level preprocessing (resize/normalize) and CPU–GPU data transfer, which is approximately 11 ms higher than the pure TensorRT inference benchmark (17 ms). The total pipeline latency (image capture → inference → visualization → publishing) is about 37 ms, where plotting bounding boxes costs about 7 ms, and ROS message serialization costs about 0.5 ms. For scenarios with stricter real-time constraints, further optimization can be achieved by calling the TensorRT engine via a C++ API or by asynchronously handling visualization, approaching the theoretical throughput limit.

**Figure 9:**
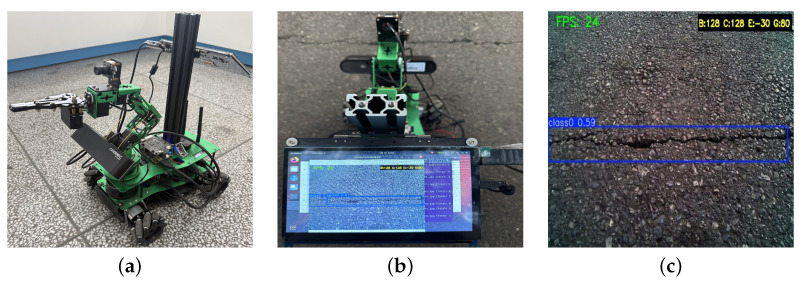
Real-world inspection on ROSMASTER X3 Plus. (**a**) Robot platform; (**b**) on-road test scene; (**c**) crack detection visualization.

Figure 9 shows the detection performance of ROSMASTER X3 Plus in real-world scenarios, verifying the engineering practicality of combining the YOLO Crack algorithm with the Jetson Orin NX platform.

#### 3.8.3. Throughput Stability Under Different Real-World Conditions

To evaluate whether the end-to-end throughput remains stable under different real-world inspection conditions, we further measured the ROS pipeline FPS under several scenes with varying lighting and background complexity. The results are shown in Table 10.

The throughput remains close to or above 25 FPS in most scenes. Slight decreases occur under complex textures and low illumination, mainly due to increased preprocessing and visualization overhead rather than changes in TensorRT inference latency. Profiling shows that pure TensorRT inference takes approximately 17 ms, whereas the complete ROS pipeline takes about 37–40 ms because of Python-level preprocessing, CPU-GPU memory transfer, bounding-box drawing, and message serialization. Therefore, further optimization can be achieved by invoking the TensorRT engine through a C++ API and asynchronously handling visualization.

## 4. Discussion

The experimental results indicate that the improvement of YOLO-Crack mainly comes from matching the detector design to the geometric properties of cracks, rather than simply increasing model capacity. Compared with generic YOLO baselines, the proposed modules provide gains with only a small increase in parameters and GFLOPs. This is consistent with recent studies showing that feature decomposition and multi-flow fusion can improve detection-related tasks under complex visual conditions [47]. However, YOLO-Crack differs from generic multi-flow designs in that its three components are tied to crack-specific failure modes: weak elongated responses, boundary attenuation during upsampling, and unstable regression for slender boxes.

First, CFCA improves crack representation by decomposing attention into channel, spatial, and directional paths. Existing SE-, CBAM-, Triplet-Attention-, or Coordinate-Attention-based methods mainly reweight generic channels or spatial coordinates, whereas CFCA explicitly introduces an axial direction branch and combines it with Sobel-guided soft edge gating. The orientation-wise ablation shows that the direction branch improves not only horizontal and vertical cracks but also diagonal cracks. This suggests that the two axial convolutions should be interpreted as lightweight directional bases for elongated local structures, not as a hard assumption that all cracks are horizontal or vertical.

Second, DFEM addresses the detail-loss problem in top-down feature fusion. Standard upsampling tends to smooth high-frequency boundaries, which is harmful for thin and low-contrast cracks. DFEM decouples semantic refinement from edge-sensitive residual enhancement, allowing the detector to preserve weak boundaries while maintaining stable semantic responses. The DFEM ablation confirms that the two-branch FRM + EE design performs better than either branch alone, while adding a third spatial-context branch introduces extra interference and does not improve detection.

Third, the smooth quality window localization adjustment contributes by aligning the regression objective with slender crack geometry while preserving the original YOLO training pipeline. Its value lies in a simple but useful empirical idea: CIoU quality is treated as a continuous optimization state, and a differentiable window term is used to calibrate the regression pressure around selected quality boundaries instead of assigning samples to hard/easy groups. This is especially relevant for crack boxes that have already captured the long-axis region but still require short-side and boundary refinement. The shape consistency and aspect ratio regularization terms further penalize scale and aspect ratio deviations, which are often under-optimized by IoU-dominated losses. These terms are implemented as weighted additions to the original YOLO localization loss, so the baseline regression objective remains dominant and training stability is preserved.

The cross-dataset and robustness results also clarify the boundary of the proposed method. YOLO-Crack consistently improves over YOLOv11n on Crack-Seg, DeepCrack, and CrackTree200, but the absolute performance on CrackTree200 remains low. This does not contradict the motivation of CFCA: the direction branch enhances local anisotropic responses, whereas CrackTree200 contains many tortuous, branching, and tree-shadow-affected cracks whose topology is difficult to represent with horizontal bounding boxes. In such cases, the limitation lies not only in feature representation but also in the detection formulation itself. Segmentation, skeleton extraction, or a cascaded detection and segmentation pipeline would be more suitable when precise crack morphology is required.

The comparison with recent crack analysis models should therefore be interpreted carefully. Many advanced crack methods are segmentation-oriented and report pixel-level metrics on different datasets, while several crack-detection studies rely on private or self-built detection datasets. Directly placing these results in the same speed-accuracy table would be methodologically unfair because the task definitions, annotation granularity, training data, and deployment hardware differ. Accordingly, this work does not claim to outperform all edge-deployable crack segmentation systems. Its contribution is a reproducible detection-oriented workflow for real-time edge screening: converting public crack masks to detection boxes, improving a lightweight YOLO detector with crack-aware modules, and validating the complete PyTorch-ONNX-TensorRT-ROS pipeline on a Jetson Orin NX robot.

Several limitations remain. First, bounding boxes provide only coarse localization and cannot describe crack width, skeletons, branch points, or curved topology. Second, RGB-only perception is vulnerable to crack-like distractors such as shadows, stains, and linear debris, as shown by the robustness experiment. Third, the current model is trained mainly on Crack500-derived detection annotations, so cross-domain performance is limited by dataset and annotation bias. Fourth, although the pure TensorRT engine reaches 58.7 FPS, the complete ROS pipeline is close to the 25 FPS real-time threshold because preprocessing, visualization, and message passing introduce additional latency. Future work will explore mixed-source training, domain adaptation, segmentation-guided supervision, multimodal sensing, and C++/asynchronous deployment to improve both robustness and system throughput.

## 5. Conclusions

This paper presents YOLO-Crack, a crack detection framework designed for real-time localization in mobile inspection scenarios. By explicitly embedding crack geometric priors into a lightweight YOLO-style detector, the proposed method achieves a favorable balance between detection accuracy and computational efficiency. Unlike conventional approaches that treat cracks as generic objects, YOLO-Crack incorporates a direction-aware attention design, a decoupled edge-preserving feature enhancement strategy, and an empirical smooth quality window localization adjustment with shape consistency supervision, enabling the network to better model the linear, slender, and anisotropic nature of crack structures.

Experimental results on Crack500 demonstrate that YOLO-Crack consistently outperforms the YOLOv11n baseline while maintaining a compact model size. Furthermore, the complete training-to-deployment workflow, including model conversion and acceleration with TensorRT and an on-robot ROS integration, verifies the practical feasibility of the proposed method under resource-constrained edge settings.

Future work may proceed in the following directions: (1) explore a cascaded architecture for detection and segmentation to address the issue of insufficient granularity in locating ultra-long cracks with detection boxes; (2) introduce multimodal data (depth information, infrared images) to enhance robustness under complex lighting conditions; (3) conduct broader cross-dataset validation and domain adaptation on datasets such as DeepCrack, CrackTree200, and Crack-Seg to evaluate the generalization performance of the method across different road surface materials and acquisition devices; (4) further enhance end-to-end throughput through system-level techniques such as asynchronous inference and multi-threading optimization.

## Figures and Tables

**Table 1:** External crack datasets used for cross-dataset evaluation.

Dataset	Description	URL
Crack-Seg	The Crack Segmentation Dataset is an extensive resource designed for individuals involved in transportation and public safety studies. Comprising 4029 static images captured from diverse road and wall scenarios, this dataset is a valuable asset for crack segmentation tasks.	https://github.com/ultralytics/assets/releases/download/v0.0.0/crack-seg.zip (accessed on 1 June 2026)
DeepCrack	The crack segmentation dataset encompasses 537 RGB color images, each with dimensions of 554 × 384 pixels. This dataset is characterized by its inclusion of images at various scales, showcasing cracks that can occur on surfaces composed of different materials. It features images depicting cracks on both concrete and asphalt surfaces.	https://github.com/yhlleo/DeepCrack (accessed on 1 June 2026)
CrackTree200	CrackTree200 offers high-resolution images at 800 × 600 pixels along with corresponding label values identifying surface cracks on asphalt surfaces. The dataset includes numerous images featuring cracks on asphalt surfaces with tree shadows.	https://github.com/fyangneil/pavement-crack-detection/tree/master?tab=readme-ov-file (accessed on 1 June 2026)

**Table 2:** Ablation study of the proposed components.

Configuration	Params	GFLOPs	P	R	mAP@.5
	(M)		(%)	(%)	(%)
Baseline (YOLOv11n)	2.58	6.30	74.6	49.7	62.8
+CFCA	2.64	6.30	77.2	50.1	64.1
+DFEM	2.67	6.80	75.0	51.1	64.0
+SQW loss	2.58	6.30	75.2	52.2	64.6
YOLO-Crack (Full)	2.73	6.85	78.8	51.4	65.7

**Table 3:** DFEM ablation on branch architectures.

Branch Design	Params	GFLOPs	P	R	mAP@.5
	(M)		(%)	(%)	(%)
Single-branch baseline	2.58	6.50	72.1	47.3	60.5
FRM only	2.61	6.65	73.5	50.3	62.8
EE only	2.58	6.50	74.8	48.9	62.5
FRM + EE (Ours)	2.67	6.80	75.0	51.1	64.0
FRM + EE + SCM	3.19	7.45	74.3	50.8	63.4

Note: All configurations use SE fusion for fair comparison. SCM is an additional spatial context branch based on a 7 × 7 depthwise convolution.

**Table 4:** Orientation-wise sensitivity analysis of the CFCA direction branch on Crack500.

Orientation	Variant	Images	Instances	P	R	mAP@0.5
				(%)	(%)	(%)
Horizontal	w/o direction branch	196	376	79.5	49.6	64.3
Horizontal	full CFCA	196	376	84.2	48.3	66.4
Vertical	w/o direction branch	46	85	52.5	49.4	52.2
Vertical	full CFCA	46	85	65.5	47.1	53.3
Diagonal	w/o direction branch	101	175	82.1	50.3	66.3
Diagonal	full CFCA	101	175	83.6	52.4	67.0

**Table 5:** Comparison with mainstream lightweight models.

Model	Params	GFLOPs	P	R	mAP@.5
	(M)		(%)	(%)	(%)
YOLOv5n	1.87	4.5	71.3	46.2	59.4
YOLOv5s	7.23	16.5	73.5	48.9	61.8
YOLOv8n	3.01	8.1	74.1	49.3	62.3
YOLOv11n	2.58	6.4	74.6	49.7	62.8
YOLO-Crack	2.73	6.85	78.8	51.4	65.7

**Table 6:** Stability validation under three random dataset splits on Crack500.

Model	Split	Images	Instances	P	R	mAP@0.5
				(%)	(%)	(%)
YOLOv11n	1	348	669	75.7	46.9	59.0
YOLO-Crack	1	348	669	78.2	50.9	63.2
YOLOv11n	2	348	626	76.1	52.1	65.7
YOLO-Crack	2	348	626	79.6	51.3	66.2
YOLOv11n	3	348	646	66.5	49.4	60.2
YOLO-Crack	3	348	646	74.7	49.8	61.7
YOLOv11n	Mean ± Std	-	-	72.8 ± 4.4	49.5 ± 2.1	61.6 ± 2.9
YOLO-Crack	Mean ± Std	-	-	77.5 ± 2.1	50.7 ± 0.6	63.7 ± 1.9

**Table 7:** Direct cross-dataset transfer results.

Dataset	Model	Images	Instances	P	R	mAP@0.5
				(%)	(%)	(%)
Crack-Seg	YOLOv11n	200	249	48.7	48.0	40.1
	YOLO-Crack	200	249	55.6	50.8	46.8
DeepCrack	YOLOv11n	237	873	45.8	26.5	37.8
	YOLO-Crack	237	873	51.4	26.9	38.2
CrackTree200	YOLOv11n	206	570	20.4	25.4	14.6
	YOLO-Crack	206	570	27.8	30.6	20.7

**Table 8:** Robustness evaluation of YOLOv11n and YOLO-Crack under challenging visual conditions.

Condition	Model	P	R	mAP@0.5	Drop
		(%)	(%)	(%)	(%)
Clean (baseline)	YOLOv11n	74.6	49.7	62.8	0.0
	YOLO-Crack	78.8	51.4	65.7	0.0
Gaussian noise σ=35	YOLOv11n	66.8	48.0	58.3	−4.5
	YOLO-Crack	70.2	49.6	62.0	−3.7
Low illumination	YOLOv11n	71.0	45.3	58.3	−4.5
	YOLO-Crack	72.8	48.0	61.7	−4.0
High illumination	YOLOv11n	76.6	46.1	61.2	−1.6
	YOLO-Crack	75.0	50.9	64.2	−1.5
Crack-like interference	YOLOv11n	48.6	45.5	41.7	−21.1
	YOLO-Crack	50.6	44.1	42.3	−23.4

**Table 9:** Inference performance on Jetson Orin NX.

Model	Precision	Latency (ms)	FPS	mAP@.5 (%)
YOLOv11n	FP32	17.38	57.5	62.8
YOLOv11n	FP16	9.95	100.5	62.8
YOLO-Crack	FP32	27.87	35.9	65.7
YOLO-Crack	FP16	17.03	58.7	65.7

**Table 10:** End-to-end throughput stability under different real-world conditions.

Scene	Lighting Condition	Background Complexity	End-to-End FPS
Campus road-normal	normal	low	26.4 ± 1.0
Campus road-shadow	uneven illumination	medium	25.2 ± 1.3
Concrete surface	normal	medium	25.8 ± 1.1
Complex texture	normal	high	24.7 ± 1.5
Low illumination	weak light	medium	24.9 ± 1.4
Overall	mixed	mixed	25.5 ± 1.2

## Data Availability

The original Crack500, Crack-Seg, DeepCrack, and CrackTree200 datasets are publicly available from their respective repositories cited in this article. The Crack500 dataset used is publicly available at https://github.com/fyangneil/pavement-crack-detection (accessed on 1 July 2025). The crack-seg dataset used is publicly available at https://github.com/ultralytics/assets/releases/download/v0.0.0/crack-seg.zip (accessed on 1 June 2026). The DeepCrack dataset used is publicly available at https://github.com/yhlleo/DeepCrack (accessed on 1 June 2026). The CrackTree200 dataset used is publicly available at https://github.com/fyangneil/pavement-crack-detection/tree/master?tab=readme-ov-file (accessed on 1 June 2026).
